# Four-dimensional experimental characterization of partially coherent light using incoherent modal decomposition

**DOI:** 10.1515/nanoph-2023-0288

**Published:** 2023-07-26

**Authors:** Xingyuan Lu, Zhuoyi Wang, Chengliang Zhao, Qiwen Zhan, Yangjian Cai

**Affiliations:** School of Physical Science and Technology, Soochow University, Suzhou 215006, China; School of Optical-Electrical and Computer Engineering, University of Shanghai for Science and Technology, Shanghai 200093, China; Shandong Provincial Engineering and Technical Center of Light Manipulations & Shandong Provincial Key Laboratory of Optics and Photonic Device, School of Physics and Electronics, Shandong Normal University, Jinan 250358, China

**Keywords:** four-dimensional characterization, incoherent modal decomposition, optical coherence, partially coherent light

## Abstract

The intensity distributions and statistics of partially coherent light fields with random fluctuations have proven to be more robust than for coherent light. However, its full potential in practical applications has not been realized due to the lack of four-dimensional optical field measurement. Here, a general incoherent modal decomposition method of partially coherent light field is proposed and demonstrated experimentally. The decomposed random modes can be used to, but not limited to, reconstruct average intensity, cross-spectral density, and orthogonal decomposition properties of the partially coherent light fields. The versatility and flexibility of this method allows it to reveal the invariance of light fields and to retrieve embedded information after propagation through complex media. The Gaussian-shell-model beam and partially coherent Gaussian array are used as examples to demonstrate the reconstruction and even prediction of second-order statistics. This method is expected to pave the way for applications of partially coherent light in optical imaging, optical encryption, and antiturbulence optical communication.

## 1 Introduction

Invariance in intensity [1], coherence [2], and polarization [3] always attracts great interest, especially when a light beam passes through a complex media. However, different from the intensity, invariance in polarization and coherence is difficult to measure. Recently, with the benefit of basis-independent measurement scheme, the polarization inhomogeneity, which defines vectorial structured light, has been proven to be invariant even in turbulence. However, for a randomly fluctuating partially coherent light field [4], the invariance in coherence structures is only found for specially designed model [2]. For general partially coherent light, the research into its propagation invariance has been hindered by the lack of genuine four-dimensional optical field measurement scheme.

The four-dimensional cross-spectral density function in the space-frequency domain, that is, the statistical average of the fluctuating electric fields [5], has been proposed for characterizing partially coherent beam. Due to the unique advantages of high signal-to-noise ratio [6–8], high-resolution imaging [9], and antiturbulence transmission [10], plenty of schemes for generating partially coherent light with novel coherence structures have been proposed, such as those based on Van Cittert–Zernike theorem [11], modal superposition [12, 13], rotating diffusers, light emitting diodes, lithium niobate [14], and disorder-engineered statistical photonic platform [15].

To characterize partially coherent light field, various methods have been proposed to measure the complex-valued cross-spectral density distribution, such as Young’s double slit [15], generalized Hanbury Brown–Twiss experiment [16, 17], diffraction through obstacles [18], and holography [19, 20]. Existing methods generally focus on measuring two-dimensional coherence structures of a certain point to the whole surface. Even the best measurement of the partially coherent light field only determines finite two-dimensional slices of coherence features [19, 20], which cannot be regarded as a genuine four-dimensional characterization of the partially coherent light field. Its importance is analogous to the difference between measuring the electric field and detecting the intensity for coherent light field. Because of the loss of information in incomplete measurements, the measurement cannot yield characteristics of the field beyond the measurement planes.

In this work, inspired by modal superposition, an experimental incoherent modal decomposition scheme is proposed and demonstrated. The flexibility of characterizing the four-dimensional partially coherent beam with decomposed random modes is shown with a Gaussian-shell-model (GSM) beam and a partially coherent Gaussian array beam. The second-order statistics, including but not limited to average intensity and cross-spectral density, can be freely reconstructed with decomposed modes. No reference arm is introduced, and no prior knowledge of light field is required. The unique advantage that distinguishes it from two-dimensional measurement is that the second-order statistic on the source plane or any other transmission planes can be retrieved with angular spectrum propagation. In addition, the interference properties predicted with the decomposed modes are highly consistent with the experimentally captured ones. This work has potential applications in the measurement of second-order statistics properties [21], coherence manipulation [22], optical imaging [23, 24], topological charge measurement [25, 26], and optical information encryption [2, 27].

## 2 Theory

Consider a scalar partially coherent source that propagates closely along the *z* axis. The second-order statistical properties of the source field can be characterized in the space-frequency domain by an electric cross-spectral density function $W\left(r_{1},r_{2}\right)=E^{*}\left(r_{1}\right)E\left(r_{2}\right)$, where the asterisk and angle brackets denote complex conjugate and ensemble average; **r**_1_ and **r**_2_ are two transverse position vectors; and $E\left(r\right)$ denotes the field fluctuating in a direction perpendicular to the *z* axis. If we can obtain the full information of cross-spectral density, the partially coherent beam can be back propagated to the source. On the other hand, the statistical properties of the partially coherent beam passing through optical system or complex media can be predicted with this knowledge as well.

Partially coherent beams can be regarded as incoherent superposition of fully coherent beams [28]. Thus, in general, one can represent the cross-spectral density function of a statistically stationary partially coherent source with orthogonal decomposition, or random modal decomposition as [12],

(1) $$W\left(r_{1},r_{2}\right)=\overset{M}{\underset{m=1}{\sum }}P_{m}^{*}\left(r_{1}\right)P_{m}\left(r_{2}\right).$$

Here, $\left\{P_{m}\left(r\right)\right\}$ represent a set of incoherent modes, where “*m*” means the *m*th mode whose total number is *M*. The cross-spectral density satisfies a pair of Helmholtz equations, and it contains information about the spectral density (i.e., average intensity) $I\left(r\right)=W\left(r,r\right)$. The Eq. (1) provides inspiration that as long as the modes $\left\{P_{m}\left(r\right)\right\}$ are measured, the complete cross-spectral density information of a partially coherent beam can be reconstructed.

**Figure 1:**
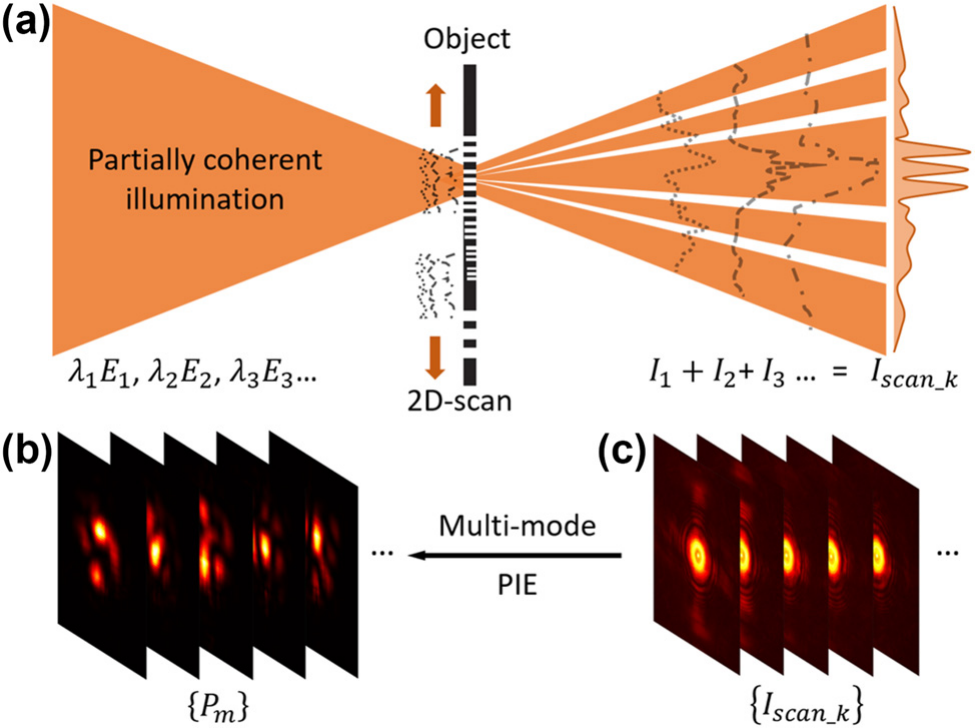
Principle and diagram of modal decomposition of a partially coherent beam. (a) A partially coherent light composed of fluctuating modes $\left\{\lambda_{j}E_{j}\right\}$ illuminates an object and the averaged diffraction patterns *I*_scan_k_ are detected by the camera. By two-dimensional scanning on the object plane, (b) a set of modes $\left\{P_{m}\left(r\right)\right\}$ are reconstructed from (c) a set of diffraction patterns $\left\{I_{scan\text{\_}k}\right\}$ with multi-mode ptychography iterative engine (PIE). Each diffraction pattern corresponds to an averaged intensity *I*_scan_k_ at each scan position.

Figure 1 shows a diagram of the diffraction for a partially coherent beam illuminating an object. The partially coherent light composed of mixed modes *λ*_1_*E*_1_, *λ*_2_*E*_2_, … *λ*_*n*_*E*_*n*_ illuminates an object $O\left(r\right)$ and the diffraction patterns *I*_1_, *I*_2_, …, *I*_*n*_ are detected and averaged to *I*_scan_k_ by the camera. Here, *λ*_*j*_ denotes the modal-weight of corresponding electric mode *E*_*j*_ [12], and *n* represents the number of fluctuating electric modes in the partially coherent light source. Based on Eq. (1), the cross-spectral density on the detection plane (far field) can be approximated as

(2) $$\begin{array}{ll} W\left(\rho_{1},\rho_{2}\right) & =F\left\{W\left(r_{1},r_{2}\right)O^{*}\left(r_{1}\right)O\left(r_{2}\right)\right\}\\ & =\sum_{m=1}^{M}F\left\{P_{m}^{*}\left(r_{1}\right)O^{*}\left(r_{1}\right)P_{m}\left(r_{2}\right)O\left(r_{2}\right)\right\}, \end{array}$$

where **ρ** is the coordinate on the detector plane and F represents Fourier transform. For other linear optical systems, Fourier transform operator can be replaced with their corresponding operators. Then, the average diffraction intensity $I_{scan\text{\_}k}\left(\rho \right)$ equals $\text{W}\left(\rho ,\rho \right)$.

Based on Eq. (2), the average diffraction intensity is highly related to the object and illumination. Thus, we introduced the multi-mode ptychography iterative engine (PIE) to reconstruct the object $O\left(r\right)$ and a set of probes $\left\{P_{m}\left(r\right)\right\}$ [29]. The partially coherent illumination scans on the object plane and covers different, but overlapping areas of the object. Then, a set of diffraction intensities $\left\{I_{scan\text{\_}k}\right\}$ can be collected. The subscript “scan_k” means the *k*th scan position. To minimize the error of exit wave, the updated probe $P_{m}\left(r\right)$, that is the mode in Eq. (1), after *i*th iteration can be written as [29]

(3) $$P_{m,k}^{i+1}\left(r\right)=P_{m,k}^{i}\left(r\right)+\beta \frac{{O^{*}}_{k}^{i}\left(r\right)}{{\left|O_{k}^{i}\left(r\right)\right|}_{max}^{2}}\left[\psi_{m,k}^{\prime i}\left(r\right)-\psi_{m,k}^{i}\left(r\right)\right].$$

Here, the update factor *β* is set as 0.9 in the following data analysis. $O_{k}^{i}\left(r\right)$ is the object at the *k*th scanning area after the *i*th iteration. $\psi_{m,k}^{i}\left(r\right)$ is the exit wave right after the object. ${\psi^{\prime i}}_{m,k}\left(r\right)=F^{-1}\left\{{\Psi^{\prime i}}_{m,k}\left(\rho \right)\right\}$, where ${\Psi^{\prime i}}_{m,k}\left(\rho \right)$ is updated complex diffraction field and can be renewed from $\Psi_{m,k}^{i}\left(\rho \right)$ with the *k*th captured diffraction intensity *I*_scan_k_ as

(4) $$\begin{array}{ll} {\Psi^{\prime }}_{m,k}^{i}\left(\rho \right) & =\left(\sqrt{I_{scan\text{\_}k}\left(\rho \right)}/\sqrt{\sum_{M}{\left|\Psi_{m,k}^{i}\left(\rho \right)\right|}^{2}}\right)\Psi_{m,k}^{i}\left(\rho \right), \end{array}$$

where $\Psi_{m,k}^{i}\left(\rho \right)=F\left\{\psi_{m,k}^{i}\left(r\right)\right\}$. To improve the reconstruction efficiency, the object is pre-reconstructed with ptychography under coherent light illumination (see Figure S3). The total number of iterations is set as 100. $\left\{P_{m}\left(r\right)\right\}$ measured by the multi-mode ptychography iterative engine are the modes on the object plane.

For any linear and time invariant system whose transmission matrix is measurable, $\left\{P_{m}\left(r\right)\right\}$ can also be inversely propagated to the source plane as $\left\{E_{m}\left(s\right)\right\}$ via angular spectrum propagation. It should be noted that the reconstructed modes $\left\{E_{m}\left(s\right)\right\}$ may not necessarily be the same as the fluctuating modes $\left\{\lambda_{j}E_{j}\right\}$ in the partially coherent light source. However, in the following analysis, we will demonstrate the effectiveness of these measured modes in reconstructing and predicting the second-order statistics and propagation characteristics of partially coherent beams.

## 3 Results

### 3.1 Experimental set-up

**Figure 2:**
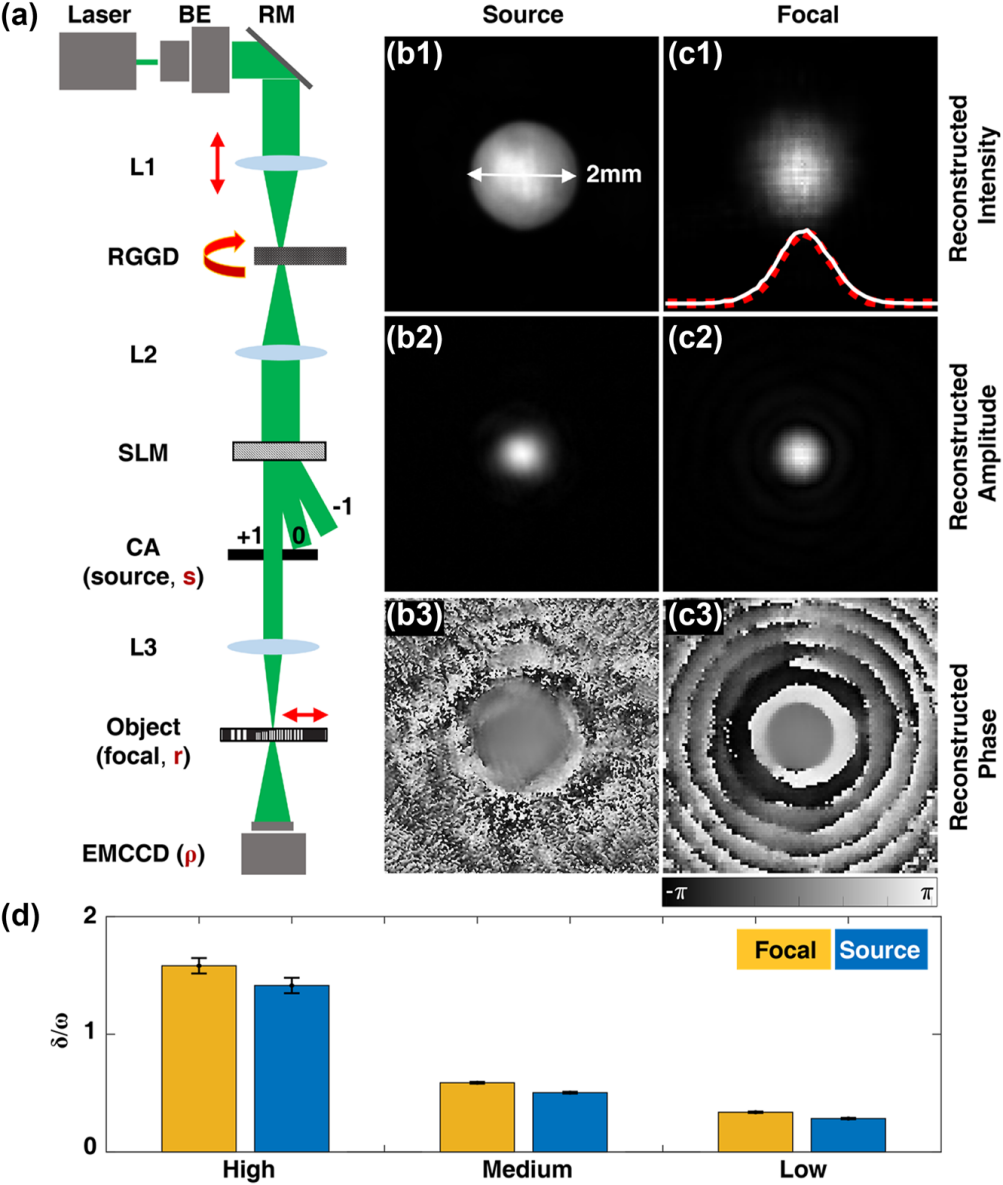
Second-order statistics reconstruction of partially coherent GSM from experimentally decomposed modes. (a) Schematic diagram of the experimental setup. BE, beam expander; RM, reflective mirror; RGGD, rotating ground glass disk; SLM, spatial light modulator; CA, circular aperture; *s*, *r*, and **ρ** denote vector coordinates on the source, object, and detection plane, respectively. Focal average intensity, cross-spectral density amplitude and cross-spectral density phase patterns of a GSM on the (b1–b3) source and (c1–c3) focal plane. Illustration in (c1) shows a fitting of actually captured intensity (white-solid curve) and reconstructed intensity (red-dashed curve). (d) Shows the reconstructed ratio of beam waist *ω* and spatial coherent width *δ* for cases with high to low coherence. The error bars represent the standard deviation of ten measurement results.

A proof-of-principle experiment was conducted with GSM beam and partially coherent Gaussian array beam. It will be demonstrated that the measured modes $\left\{P_{m}\left(r\right)\right\}$ can be used to reconstruct the second-order statistics, including intensity and cross-spectral density. The schematic diagram of the experimental setup is shown in Figure 2(a). Partially coherent light source is generated with coherent laser beam and a rotating ground glass disk based on Van Cittert–Zernike theorem [11, 30]. The initial spatial coherence width *δ*_0_ of the collimated beam after lens L2 is determined by the focal spot size on the rotating ground glass disk (RGGD) and the roughness together. The lens (L1) can be shifted back and forth to realize the size control of focal spot. The larger the beam spot is, the less coherent the generated partially coherent beam will be. A spatial light modulator (SLM) was used to modulate the amplitude profile of the output partially coherent beam. Then, a GSM or a partially coherent Gaussian array beam can be generated as partially coherent light source shown in Figure 1(a).

Taking the back focal plane of lens L3 as the region of interest, an object (USAF resolution test chart) is placed perpendicular to optical axis, after which the diffractive intensities are captured with a camera (EMCCD, iXon Life, Oxford). The distance of the object to the camera is set as 146 mm. In order to collect more diffraction information, overlap scanning is performed by fixing the object on a two-dimensional mobile stage (CONEX-MFACC Newport). A total of 400 raw diffraction patterns were captured via 20 × 20 two-dimensional (2D) overlap scanning. The probe beam width is around 200 μm, and the shifting step size is set as 40 μm. After 100 iterations, a set of probes $\left\{P_{m}\left(r\right)\right\}$ can be reconstructed with Eqs. (3) and (4), and these probes are the final decomposed modes of the partially coherent light on the focal (object) plane. In the following analysis, *s*, *r*, and **ρ** denote vector coordinates on the source, focal (object), and detection plane, respectively.

To demonstrate the modal decomposition of a GSM beam, a Gaussian amplitude mask with beam waist *ω*_0_ = 1 mm is loaded on the SLM. The theoretical model of a GSM on the source plane is $W\left(s_{1},\;s_{2}\right)=\exp\left[-\left(s_{1}^{2}+s_{2}^{2}\right)/4\omega_{0}^{2}-{\left(s_{2}-s_{1}\right)}^{2}/2\delta_{0}^{2}\right]$, where *ω*_0_ and *δ*_0_ are the initial beam waist and coherence width, respectively. A hard-edged aperture function with 2 mm diameter is also applied to limit the area to be reconstructed. Propagating to the focal plane, the cross-spectral density becomes $W\left(r_{1},\;r_{2}\right)=\exp\left[-\left(r_{1}^{2}+r_{2}^{2}\right)/4\omega^{2}-{\left(r_{2}-r_{1}\right)}^{2}/2\delta^{2}\right]$, where *ω* and *δ* are the beam waist and coherence width on the focal plane, respectively.

From 20 × 20 diffraction patterns (examples shown in Figure 1(c)), in total 64 random modes (examples shown in Figure 1(b)) are reconstructed and $W\left(r_{1},r_{2}\right)=\sum_{m=1}^{64}P_{m}^{*}\left(r_{1}\right)P_{m}\left(r_{2}\right)$ is computed. Figure 2(b1) shows the calculated focal intensities by modal superposition, which gives $I\left(r\right)=W\left(r,r\right)$. The fitting of actually captured focal intensity (white-solid curve) and reconstructed focal intensity (red-dashed curve) is illustrated in Figure 2(c1). To further show a two-dimensional slice of the four-dimensional cross-spectral density, we chose an on-axis reference point ($r_{2}=\left[0,\;0\right]$). The amplitude and phase of the cross-spectral density are shown in Figure 2(c2 and c3). Both the distributions of intensity and cross-spectral density agree well with the theoretical model, which obeys the Gaussian distribution. The rings around the cross-spectral density amplitude central spot and the ring dislocations in the phase are caused by the hard-edge aperture on the source.

A focusing lens can be regarded as a linear and time invariant system and the transmission matrix is measurable. Thus, the modes $\left\{P_{m}\left(r\right)\right\}$ can be inversely propagated to the source plane as $\left\{E_{m}\left(s\right)\right\}$ via angular spectrum propagation. Then $W\left(s_{1},s_{2}\right)=\sum_{m=1}^{64}E_{m}^{*}\left(s_{1}\right)E_{m}\left(s_{2}\right)$ and $I\left(s\right)=W\left(s,s\right)$. As shown in Figure 2(b1), the boundary of the hard-edge aperture can be clearly observed, whose size is 2 mm in diameter. It means the source intensities calculated with $\left\{E_{m}\left(s\right)\right\}$ also agree very well with the ground truth. Furthermore, the cross-spectral density amplitude distribution in the source plane also presents a Gaussian profile, as shown in Figure 2(b2), and the cross-spectral density phase shown in Figure 2(b3) is nearly uniform, because there is no phase term in the cross-spectral density function of GSM.

As the initial coherence width decreases, the focal and source intensities maintain Gaussian profile, while the focal beam waist will increase gradually. Figure 2(d) shows the measured ratio of coherence/beam-waist (that is *δ*/*ω*) for different levels of coherence (high, medium, and low). Ten datasets are analyzed for each case and the height of each bar is set as the mean value of ten measurements. It shows that, the source ratio is nearly the same as that on the focal plane, which is consistent with the theoretical prediction. The error bar shows the measurement error will decrease as the coherence decreases. The slight increase from source to focal plane is caused by the aperture applied on the source GSM.

### 3.2 Inverse propagation of complex partially coherent light

**Figure 3:**
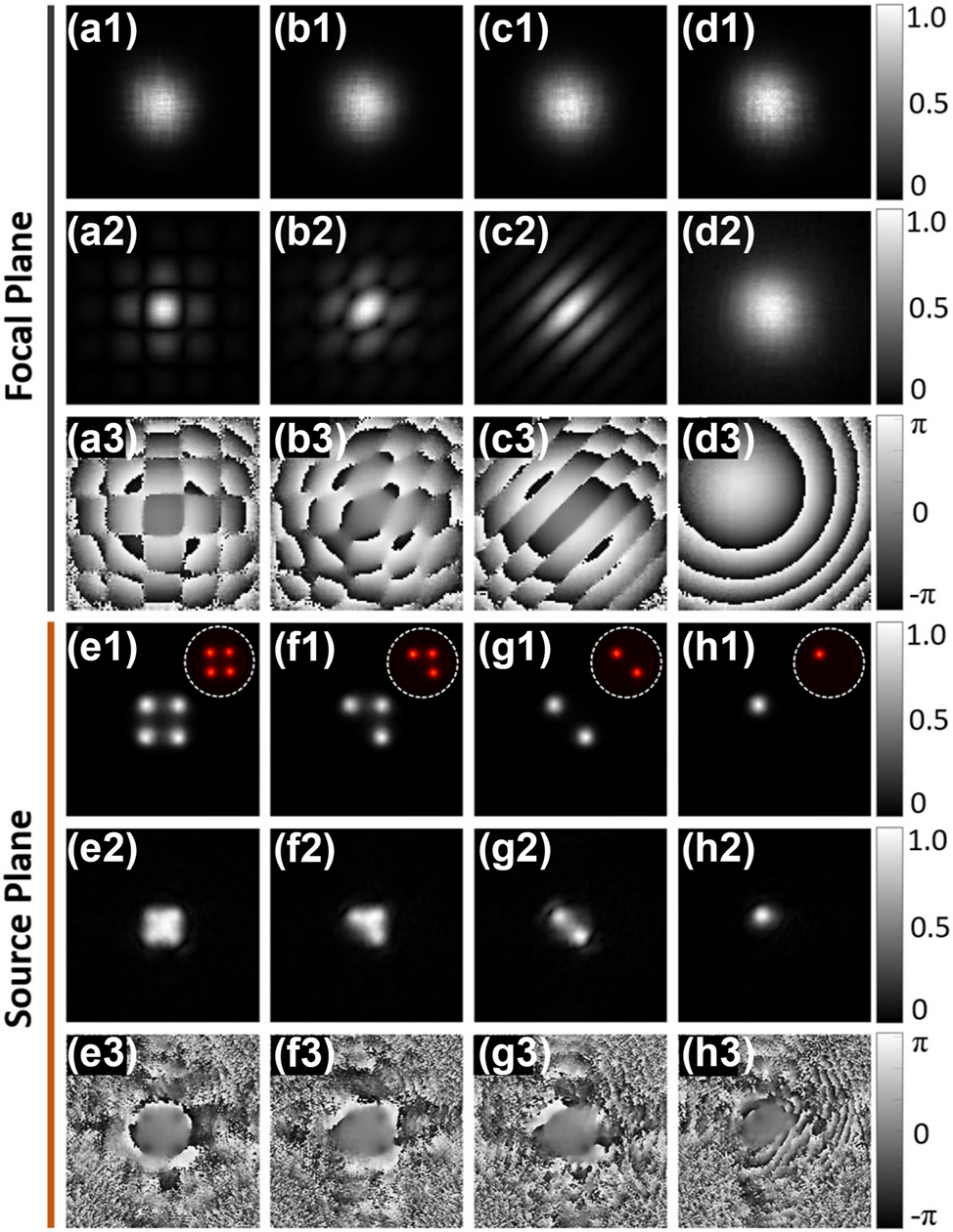
Second-order statistics reconstruction of low coherence Gaussian array from experimentally decomposed modes. (a1–d1) Focal average intensity, (a2–d2) cross-spectral density amplitude, and (a3–d3) cross-spectral density phase are reconstructed with measured focal modes $\left\{P_{m}\left(r\right)\right\}$. (e1–h1) Source average intensity, (e2–h2) amplitude, and (e3–h3) phase of cross-spectral density are reconstructed by source modes $\left\{E_{m}\left(s\right)\right\}$, which are calculated from focal modes via inverse propagation. Illustrations in (e1–h1) are the actually captured intensities on the source plane.

To further evaluate the generality of the proposed measurement method, the second-order statistics for a partially coherent Gaussian array beam [31] are measured, as shown in Figure 3. The Gaussian array mask is loaded on the SLM, and the modulated +1 order is then selected with a circular aperture (or a 4f lens system). The beam waist of each Gaussian spot is 0.3 mm and the center-to-center distance is set as 0.8 mm. Four kinds of array layouts are shown here. Each focused partially coherent Gaussian array beam illuminates the object plane, and their corresponding modes are measured. Similar with previous calculation, the intensity and cross-spectral density are reconstructed with these measured modes. It can be found that, the focal properties (Figure 3(a–d)) evolve into distributions totally different from the source plane (Figure 3(e–h)).

For high coherence light source, the focal pattern differs as the Gaussian array changes due to the interference. However, as the coherence decreases, all these focal intensities tend to be Gaussian distributed, as shown in Figure 3(a1–d1). Different from GSM beam, the cross-spectral density of partially coherent Gaussian array is complex valued, whose structure has more features. For the single Gaussian spot case, the focal intensity and cross-spectral density amplitude are similar with that shown in Figure 2, while the phase shows difference due to the off-center of Gaussian spot on the source plane. Furthermore, the intensity and cross-spectral density information on the source plane are also calculated by inverse propagation of focal plane modes to the source plane. The actually captured intensities are shown in illustrations (white dashed circle) of Figure 3(e1–h1). Obviously, the Gaussian array structures are reconstructed very well, even the focal intensity has no more features to distinguish the array layout. The proposed method is also effective for more complicated light fields, such as vortex beams with different degrees of coherence (Supplementary Figure S1). This study may find applications in optical encrypted transmission.

### 3.3 Prediction of interference properties

**Figure 4:**
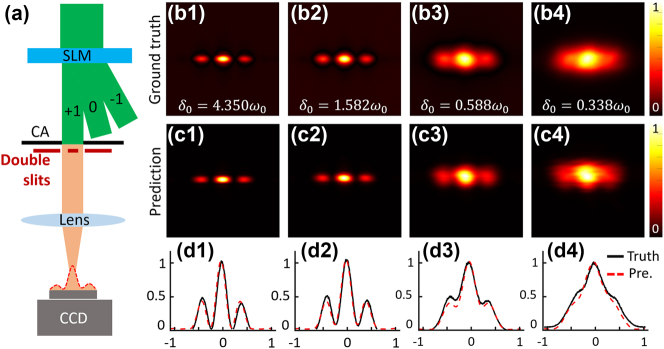
Interference prediction of a GSM beam passing through a double-slit. (a) Diagram of double-slit interference. (b1–b4) Experimentally captured interference fringes of a GSM beam passing through a double-slit under different spatial coherence. (c1–c4) Corresponding prediction results with reconstructed source modes. (d1–d4) Cross lines of predicted intensity (red dotted lines) and real-captured intensity (black lines).

After reconstructing the source modes $\left\{E_{m}\left(s\right)\right\}$ via inverse propagation of the focal plane modes $\left\{P_{m}\left(r\right)\right\}$, the interference properties of a GSM passing through a double-slit are predicted. The double-slit is located behind the circular aperture (CA), with a slit width of 0.2 mm and slits center-to-center distance of 0.4 mm. Figure 4(b1–b4) show the captured interference patterns (taken as ground truth) under different coherence widths, and Figure 4(c1–c4) show corresponding predicted interference patterns. The one-dimensional intensity fitting curves are shown in Figure 4(d1–d4). Obviously, the predicted results agree well with the ground truth. More importantly, such capability opens completely new opportunities for the future applications that requires close loop iterative control of partially coherent light propagation.

## 4 Discussions

The solution of the incoherent decomposition for a four-dimensional partially coherent light field is not unique. Thus, the correctness evaluation criterion is not the single mode itself, but the recovered statistical properties using all the modes, such as intensity and cross spectral density. The main influence of the correctness is the number of modes used in the reconstruction. Supplementary Figure S1 shows the relationship between the correlation coefficient of the reconstruction results relative to the truth and the number of modes used for reconstruction. Meanwhile, the oversampling ratio defined with the ratio of known pixels (e.g., 400 diffraction patterns) over unknown pixels (e.g., 64 complex-valued modes to be reconstructed) larger than 2 is the basic condition to be followed. Otherwise, the correlation coefficient will not increase even the number of modes increase. In other words, another solution to improve the correctness is using higher scattering objects. When choosing the scattering object, the purpose should be improving the richness of the diffraction patterns to ensure the oversampling ratio is larger than 2 [32, 33].

Theoretically, when the degree of coherence is reduced, the number of modes (*M*) will increase [12]. In the simulation of GSM modal superposition, when M equals 25 for high coherence light source and *M* equals 64 for lower coherence cases, the superimposed intensity and cross-spectral density matches well with that calculated with GSM Mathematica model. The experimental reconstruction error metric (commonly called *R* factor) can be calculated with the captured intensity *I*_scan_k_ and the calculated intensity $\sum_{M}{\left|\Psi_{m,k}^{i}\left(\rho \right)\right|}^{2}$ as

(5) $$R=\left(\sqrt{I_{scan\text{\_}k}\left(\rho \right)}-\sqrt{\underset{M}{\sum }{\left|\Psi_{m,k}^{i}\left(\rho \right)\right|}^{2}}\right)/\sqrt{I_{scan\text{\_}k}\left(\rho \right)}.$$

## 5 Conclusions

To summarize, experimental modal decomposition of partially coherent beam has been demonstrated via scanning an object with the beam to be measured. This method provides a flexible and high-precision way to fully characterize the second-order statistical properties of randomly fluctuated partially coherent beams. This scheme integrates all the functions of the existing measurement schemes of partially coherent beam, while the experimental setup is uniaxial. Only a scattering object and a camera are needed. In addition, the decomposed modes can be inversely propagated to the source plane, or any other transverse planes of a linear and time invariant optical system, as long as the transmission matrix is known or measurable. Therefore, the statistical properties of un-occurring diffraction or interference can be predicted.

In this work, we designed several experiments to demonstrate the effectiveness of the measured modes in characterizing or predicting the second-order statistics, e.g., intensity and cross-spectral density. In addition, these decomposed modes are expected to play important roles in practical application scenarios. For low coherence beam whose intensity contains few information or the beam whose information is hidden in the source cross-spectral density distribution, the modal decomposition scheme demonstrated in this work is powerful and indispensable. It has potential applications in the characterization of the superimposed partially coherent beam with complex-valued screens, such as modal-weight measurement by orthogonal decomposition. Then, the encryption of partially coherent light will become implementable. The idea of iterative reconstruction is also expected to be feasible in designing minimal-mode partially coherent light source in micro–nano system where lithium niobate crystal [34] or meta-surface [15] could be used. Furthermore, the results in Figure 4 provide inspiration that the evolution features of partially coherent light fields, which initially require three-dimensional scanning to be reconstructed, can be transformed into two-dimensional scans on a single plane. It also paves the way for the study of invariance in partially coherent beams after propagating through a complex media, such as turbulence atmosphere.

## Supplementary Material

Supplementary Material Details
