# Supplementary material for: Four-dimensional experimental characterization of partially coherent light using incoherent modal decomposition
Source: Nanophotonics. 2023 Jul 26;12(17):3463–70. doi: 10.1515/nanoph-2023-0288 (PMC11501528; doi:10.1515/nanoph-2023-0288)
Supplement: Supplementary file 1 — Supplementary Material Details [file j_nanoph-2023-0288_suppl_001.docx]

Supplementary material

Four-dimensional experimental characterization of partially coherent light using ‎incoherent modal decomposition

Xingyuan Lu,^1, ‡^ Zhuoyi Wang,^1, ‡^ Qiwen Zhan,^2,^ * Yangjian Cai,^3,^ * and Chengliang Zhao^1,^ *

^1^School of Physical Science and Technology, Soochow University, Suzhou 215006, China

^2^School of Optical-Electrical and Computer Engineering, University of Shanghai for Science and Technology, Shanghai 200093, China

^3^Shandong Provincial Engineering and Technical Center of Light Manipulations & Shandong Provincial Key Laboratory of Optics and Photonic Device, School of Physics and Electronics, Shandong Normal University, Jinan 250358, China

^‡^These authors contributed equally to this work.

[*qwzhan@usst.edu.cn](mailto:*qwzhan@usst.edu.cn) (Q. Zhan)

[*yangjian_cai@163.com](mailto:*yangjian_cai@163.com) (Y. Cai)

*zhaochengliang@suda.edu.cn (C. Zhao)

S1. Experimental results for partially coherent vortex beam.

In addition to the GSM and partially coherent Gaussian array beam, the proposed method is also effective for more complicated light fields, such as vortex beams with different degrees of coherence, as shown in Fig. S1.


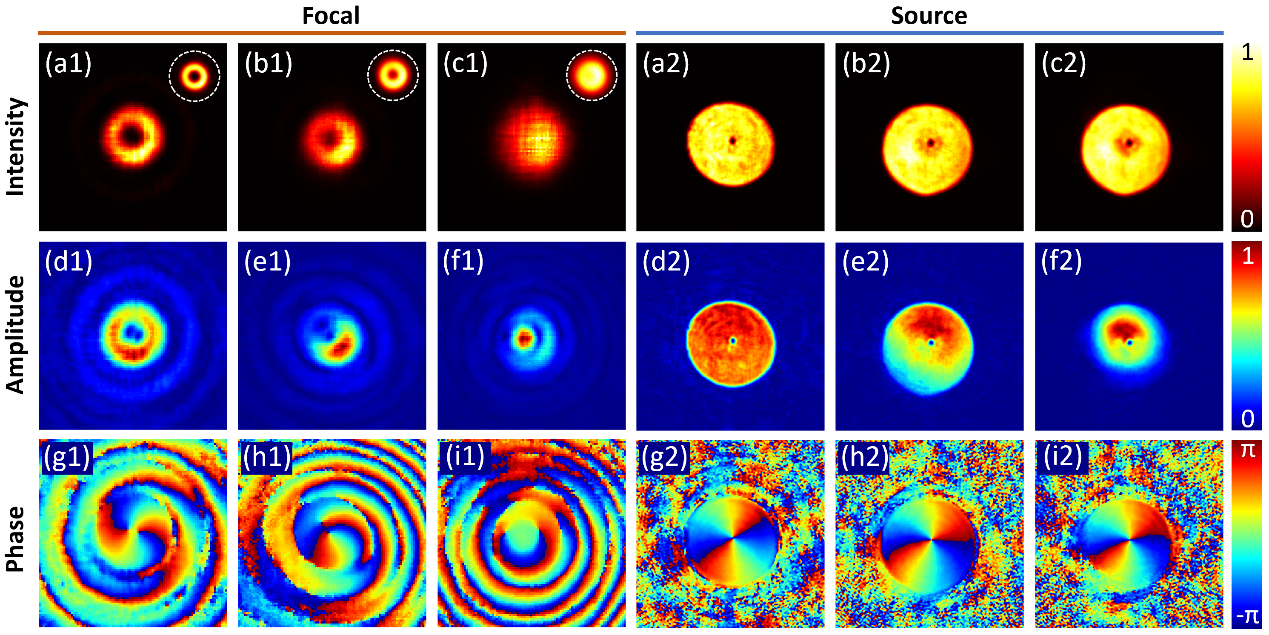


**Fig. S1.** Fig. S1. Second-order statistics reconstruction from experimentally decomposed modes of vortex beams with different degrees of coherence. (a1-c1) Focal average intensity, (d1-f1) amplitude and (g1-i1) phase of cross-spectral density are reconstructed with measured focal modes. (a2-c2) Source average intensity, (d2-f2) amplitude and (g2-i2) phase of cross-spectral density are reconstructed by source modes which are calculated from focal modes via inverse propagation. Illustrations in (a1-c1) are the actually captured intensities on the focal plane.

S2. The number of modes used in reconstruction.

The correctness evaluation criterion is not the single mode itself, but the recovered statistical properties using all the modes, such as intensity and cross spectral density. The main influence of the correctness is the number of modes used in the reconstruction. Fig. S1 shows the relationship between the correlation coefficient of the reconstruction results relative to the truth and the number of modes used for reconstruction. When the number of modes is larger than 49, e.g. 64, the correlation coefficient between the reconstruction results and the truth tends to stabilize.


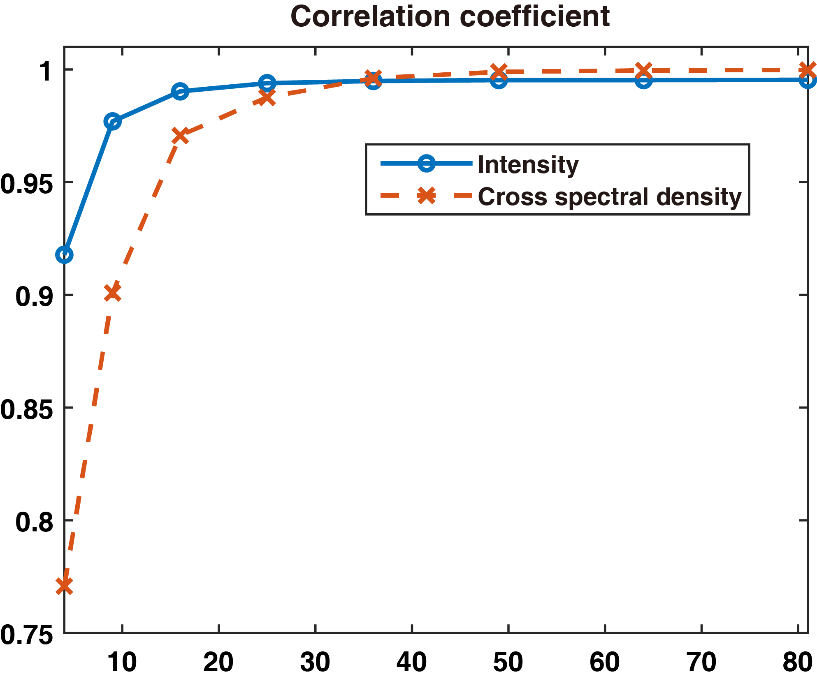


**Fig. S2.** The dependence of the correlation coefficient between the reconstruction results and the truth on the number of modes used for reconstruction.

S3. The reconstruction of the object.


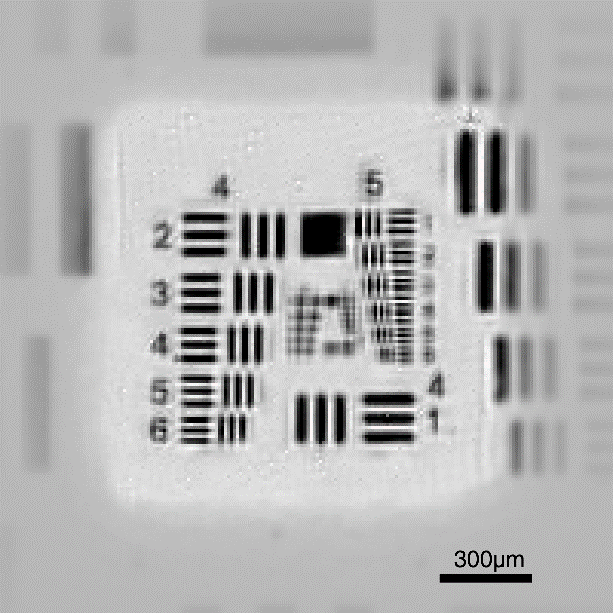


**Fig. S3.** The reconstruction of the object.
